# Viral communities of the human gut: metagenomic analysis of composition and dynamics

**DOI:** 10.1186/s13100-017-0095-y

**Published:** 2017-10-03

**Authors:** Varun Aggarwala, Guanxiang Liang, Frederic D. Bushman

**Affiliations:** 1 Department of Microbiology, University of Pennsylvania School of Medicine, 3610 Hamilton Walk, Philadelphia, PA 19104-6076 USA; 2 Division of Gastroenterology, Hepatology, and Nutrition, Children’s Hospital of Philadelphia, Philadelphia, PA 19104-4319 USA

**Keywords:** Virus, Bacteriophage, Virome, Microbiome, Metagenomics, DNA, Transduction

## Abstract

**Background:**

The numerically most abundant biological entities on Earth are viruses. Vast populations prey on the cellular microbiota in all habitats, including the human gut.

**Main body:**

Here we review approaches for studying the human virome, and some recent results on movement of viral sequences between bacterial cells and eukaryotic hosts. We first overview biochemical and bioinformatic methods, emphasizing that specific choices in the methods used can have strong effects on the results obtained. We then review studies characterizing the virome of the healthy human gut, which reveal that most of the viruses detected are typically uncharacterized phage - the viral dark matter - and that viruses that infect human cells are encountered only rarely. We then review movement of phage between bacterial cells during antibiotic treatment. Here a radical proposal for extensive movement of antibiotic genes on phage has been challenged by a careful reanalysis of the metagenomic annotation methods used. We then review two recent studies of movement of whole phage communities between human individuals during fecal microbial transplantation, which emphasize the possible role of lysogeny in dispersal.

**Short conclusion:**

Methods for studying the human gut virome are improving, yielding interesting data on movement of phage genes between cells and mammalian host organisms. However, viral populations are vast, and studies of their composition and function are just beginning.

## Background

The human virome is overwhelmingly composed of unstudied bacterial viruses of unknown importance to health and disease. Here we overview metagenomic methods for studying these populations, and some recent results.

## Main text

### Introduction

Global viral populations are vast. Rich sea water typically harbors 10^6^ bacterial cells per ml, but virus-like particles (VLPs) outnumber cells by a factor of ten [1–3]. Given the enormous number of VLPs, it is generally impossible to determine how many really correspond to infectious viruses. However, Electron microscope (EM) analysis shows that many have morphologies resembling bacterial viruses [2, 3], so it seems likely that most VLPs are real viruses. The viral populations living in healthy humans are also enormous. The human microbiome contains roughly 100 trillion cells, equaling or exceeding the number of human cells comprising our bodies [4]. Stool from healthy individuals can contain ~10^11^ cells per gram, which are predominantly bacteria, but also contain archaea and microeukaryotes [5–9]. Studies are just beginning on the viral populations associated with our microbiota, but early work has established that the communities are large and dynamic [10–19].

**Fig. 1:**
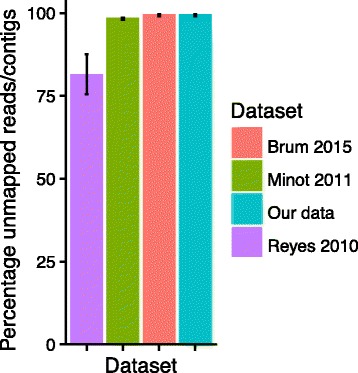
Illustration of the viral dark matter problem. Percentage of unmapped reads or contigs in several viral purified sequencing studies and on 849 viral purified sequencing datasets collected locally at University of Pennsylvania

Here we review recent studies of the human virome. Several excellent reviews have summarized a variety of aspects (e. g. [11, 20–24])—here we first review techniques for purifying viral particles, emphasizing that different methods yield different parts of the viral population. We then review bioinformatic pipelines for analyzing the output, focusing on strengths and weakness of current technology. We particularly emphasize the challenges posed by the “viral dark matter” [11, 25] —in metagenomic studies of the human virome, the vast majority of reads cannot be annotated into functional or taxonomic categories (Fig. 1). This is likely because of the enormous size and diversity of global viral populations, and the fact that only a few thousand viral genomes (7321 from NCBI Genome) are available in databases, so that any new virus captured from nature will usually not have much resemblance to a database entry. Following the review of methods, we summarize a few recent studies that illuminate the nature of the human gut virome and transfer of phage DNA sequences between cells and between humans.

### Biochemical methods for purifying and sequencing VLP genomes

It is possible to study the viral populations of human gut by purifying DNA from total stool, then sequencing and aligning the reads to viral databases [26]. However, viral DNA represents only a small minority of the total DNA recovered, and most viral sequences do not closely resemble viral genomes available in databases (the dark matter problem mentioned above) [10, 11, 15]. To provide a more comprehensive picture, it is often useful to isolate VLPs first from the sample, and then analyze the viral metagenome de novo in the sample of interest [27].

The methods used for viral particle purification have a strong effect on the populations recovered. An investigator must decide whether they want to study viral genomes made of DNA, RNA or both, and whether they want to study both enveloped and non-enveloped viruses.

In a typical protocol, feces are suspended in a buffer, and then filtration or centrifugation steps are added to remove bacterial or human cells and any particulate material [27]. Protocols vary in the amount of starting material required (0.1 g to 5 g) [10, 12–15, 28], buffers used (saline-magnesium (SM) buffer [10, 13–15]; phosphate-buffered saline (PBS) buffer [17, 29], and filter pore size. Commonly used are 0.2 and 0.45 μm, but some phages and eukaryotic viruses are larger than 0.2 μm [30]. Going the other way, bacteria smaller than 0.45 μm have been reported, so the larger pore size may result in sporadic bacterial contamination [30]. Following filtration, protein purification filters, such as Centricon Plus-70 Centrifugal Filter (Millipore) are often used for further purifying and concentrating VLPs [31]. As an alternative, cesium chloride (CsCl) density gradient centrifugation, can be used for further VLPs purification and enrichment [14, 15]. A recent study reported that including a CsCl density gradient step was better than other methods in removing host-derived DNA [30]. However, this method is time intensive, which limits the number of samples that can be processed in parallel [30].

Chloroform can be added to disrupt the cell membrane, allowing further removal of microbial and host cells and debris [14, 15, 17]. However, a disadvantage is that enveloped viruses will also be removed, and there may be other effects on viral populations as well. Thus, some researchers choose not to treat VLP preps with chloroform. This allows a more comprehensive assessment of the viruses present, but also results in more contamination with nucleic acids from cells and cellular debris, usually meaning that downstream bioinformatic steps must be relied on to distinguish viral sequences from background. The differences among methods are summarized in Table 1.

**Table 1:** Methods for purifying VLPs

VLPs isolation steps	Methods	Pros	Cons	References
Starting material amount	0.5 ~ 5 g	Recovery of low abundant viruses	Long processing time; Difficult in filtration with high mucus samples (such as meconium)	[10, 12–15]
	0.1 ~ 0.3 g	Simple and quick	Lost of low abundant viruses	[17, 28, 29, 31]
Suspension buffer	SM buffer	Long-term storage of viruses		[10, 12–15, 28, 31]
	PBS buffer			[17, 29]
Filtration pore size	0.20 μm	Better efficiency of removing host and other microbial cells	Lost of viruses larger than 0.20 μm	[13–15, 31]
	0.45 μm	Recovery of viruses larger than 0.20 μm	Less efficiency of removing host and other microbial cells	[12, 29]
	0.45 μm filtration followed by 0.20 μm filtration			[10, 17, 28]
VLPs enrichment	Centricon Centrifugal Filter	Simple and quick	Proteins from host or other microbial cells cannot be filtered	[13, 31]
	CsCl density gradient centrifugation	Better efficiency of removing host and other microbial cells	Long processing time; Limited number of samples that can be processed in parallel	[10, 14, 15]
Further purification	Usage of chloroform	Better efficiency of removing host and other microbial cells	Lost of enveloped viruses	[10, 12–15, 17, 28, 31]
	No chloroform	Recovery of enveloped viruses	Less efficiency of removing host and other microbial cells	[29]

After VLPs are isolated, free nucleic acids are removed by treating VLPs with DNase and RNase. The viral DNAs and RNAs can then be extracted by any of several methods, including standard phenol-chloroform methods [10, 12], Trizol-based methods [32], or commercial kits, such as DNeasy (Qiagen) [13, 15], or QIAmp Ultrasens Virus kit (Qiagen) [33].

The yield of nucleic acids extracted from VLPs is usually low, necessitating an amplification step before sequence analysis. A common method for DNA samples is multiple displacement amplification (MDA), which uses the highly processive phage phi29 DNA polymerase primed with random oligonucleotides to amplify viral genomes. A disadvantage of MDA is that it will preferentially amplify small circular viruses by rolling circle amplification [34]. For analyzing RNA viruses, VLP RNA must first be reverse transcribed into cDNA, then amplified by sequence-independent, single-primer amplification (SISPA) [35]. or other method [33].

After obtaining sufficient amounts of nucleic acids, virome library construction is similar to standard metagenomic library construction. For example, Illumina Nextera XT Sample Prep kit, which requires only tiny amount of starting materials, is relatively quick, though we note that recovery is not perfectly even—for example, end sequences are typically recovered inefficiently. The Illumina MiSeq and HiSeq platforms are commonly used for virome sequence analysis.

### Wrestling with contamination

Contamination is a challenge when performing metagenomic analysis of samples with low microbial biomass [36, 37]. DNA contamination can come from the laboratory environment, and from commercial reagents. Several studies have characterized the background originating in commercial reagents, and further reported that different kits can bring in different contaminants [36, 37]. Recent studies reported a large number of apparent virus-derived reads from negative control samples in studies of lung bronchoalveolar lavage, serum [33] and feces [31]. In Kim et al. [36], the authors reported numerous reads in a negative control sample which mapped to the phi29 polymerase gene--phi29 polymerase was used to perform GenomiPhi DNA amplification of the samples, suggesting that these reads are likely contamination from the phi29 polymerase protein preparation [36] (i.e. the gene used to manufacture a commercial polymerase came through in the polymerase prep!). Environmental and reagent contamination can be suppressed using ultraclean reagents, but some contamination is probably unavoidable, so it is crucial to use appropriate negative control samples to characterize the background and incorporate results into the interpretation.

### Approaches for analyzing data from virome sequencing studies

**Fig. 2:**
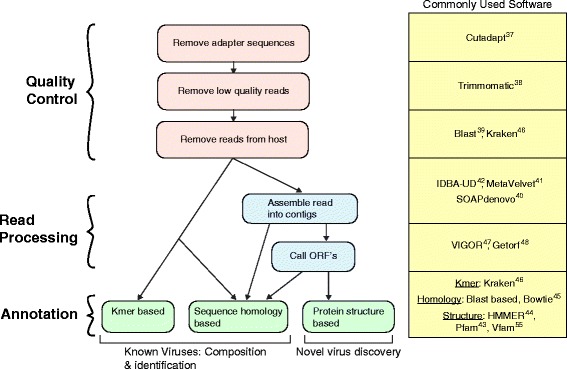
Bioinformatic approaches for analyzing the virome sequencing data. The left panel describes the steps in the analysis of the data, while the right panel lists some commonly used tools for performing the corresponding step on the left

Several approaches have been used for analyzing high throughput virome sequence data to identify the composition and types of known viruses and to discover novel viruses. The two approaches involve common steps at the start (Fig. 2). The first step involves removing the adapter sequences which were added during the library preparation stage, using, for example Cutadapt [38]. Next, low quality reads are removed using Trimmomatic [39] or custom scripts. Human reads can then be filtered out using BLAST [40].

Sequence reads can be analyzed individually, or assembled [41–43] into larger “contigs” that represent viral genomes or parts of genomes. The longer contigs provide a longer sequence for similarity searches using BLAST or motifs in inferred protein sequences using Pfam [44, 45]. Use of contigs also allows for more sensitive tracking of viruses over multiple sampling points. Methods for constructing contigs are still being optimized, and multiple challenges remain [46]. For example, sequence heterogeneity and relative abundance of genomes can affect the outcome. Downstream, BLAST [40], Bowtie [47], and Kraken [48] can all be used to detect sequence homology of reads and contigs to reference sequences in the viral database and thus quantify abundance and composition. Open reading frames (ORFs) can also be called [49, 50] on contigs to predict and identify viral genes of interest.

The NCBI Genome database includes the reference whole genome sequences of 7321 viruses. In addition, viral protein sequences are available in Refseq [51], UniProt [52], and custom databases of viral proteins are also available for VLP samples from ocean [53], various geographical habitats [54] or humans [17]. However, alignment to these databases is often challenging when sequence identity is less than 30%. Viruses often accumulate substitutions at high rates [55]--RNA viruses replicate using error prone RNA-dependent RNA polymerases [56], retroviruses use error prone reverse transcriptases [57] and single stranded DNA viruses also show high rates of substitution [55].

These challenges can be addressed by focusing on profile methods for detecting distant homologs of known viral families. The profile methods, specifically those based on hidden markov models (HMM) [45], learn position specific features from sequences and allow for variation at each site under a probabilistic framework. This allows for the query sequence to match the viral family profile HMM if it is evolving like other members in the family, even if it is not highly pairwise similar to any. Here, popular approaches include the protein family database Pfam or virus specific protein family database Vfam [58]. However, Pfam captures only 20% of viral protein families so will not annotate most viral ORFs in a sample. Vfam provides a set of HMMs derived from viral proteins, but does not have detailed annotation of protein function. Thus, further development of these tools would be useful.

Several pipelines [59–64] are available which combine different tools for pre-processing, assembly and annotation. They provide a single step portal for analysis of reads from virome sequencing datasets, using multiple available programs.

None of these tools solve the problem of the viral dark matter (Fig. 1). This is expected given the vast number of viruses in the world and the limited size of available databases. This problem is of less concern for identification and discovery of pathogenic viruses that infect human cells, where there are fewer different types, and these viruses have been closely studied because of their medical importance. However, any study focusing on phage and bacterial dynamics is greatly complicated by the dark matter problem.

### Metagenomic studies of the gut virome

In the sections below, we first review studies that begin to outline the structure of the gut virome and some aspects of its dynamics. Given the interests of the readers of Mobile DNA, we then review two topics on phage mobilization. We first review movement of medically relevant genes between bacterial cells by phage. We focus on a controversy on whether phages are or are not major vehicles for moving antibiotic resistance genes between cells. We then review metagenomic studies documenting movement of whole populations of phages between human individuals during fecal microbial transplantation.

### Composition of the human gut virome

Multiple studies have now investigated the composition of the human gut virome, providing an initial picture of its structure (e. g. [10–17, 28, 31, 65]). As above, researchers have first purified VLPs, then acquired DNA sequence data, allowing assembly and evaluation of contigs. This sketches out aspects of the viral population structure, but a complication is the fact that different viruses are present in distinct abundances. As a result, the most abundant genomes will be sequenced to greater depth, while the rarer genomes will be sparsely covered, or not represented at all. For genomes that are sparsely sequenced, read coverage will be patchy, so that rarer genomes may be represented by multiple contigs, each a fragment of the full genome. Investigators report the number of viral contigs detected, but this is a mixture of full viral genomes and fragments, so the true number of viral variants is challenging to evaluate even roughly. In another approach, the PHAACS program [66] queries how often viral reads assemble together, and uses this to estimate the number of different types. Estimates of human gut populations by PHAACS range from ~2300 to ~8000 phage genotypes. However, implementing this approach requires estimating the mean and variance in genome sizes, which is usually unknown, complicating the analysis.

A simple means of estimating viral abundance is to purify viruses from a weighed amount of stool, then stain with SYBR Gold, which binds nucleic acids, allowing counting of particles. This of course measures all types of viruses as a pool. Such counts are valuable, but we find that RNA virus stain less brightly (unpublished data), and the analysis relies on the premise that all viruses were successfully extracted from a stool sample, both significant limitations. For human stool, counts tend to range from 10^8^ to 10^9^ per gram [67] (our unpublished data); for comparison, the bacterial counts range from 10^10^ to 10^11^ [68].

Although most viral reads find no attribution of any kind, the minority that do find annotation after alignment to databases allows a provisional accounting of the viral types present. In human stool, the predominant forms are nonenveloped DNA bacteriophages. Tailed phages such as Sipho-, Podo- and Myoviridae are consistently abundant. Microviridae, non-tailed single stranded DNA phages, are also notably abundant, but these are preferentially amplified using MDA (Genomiphi), so that their true abundance in the starting sample is usually unclear without follow up studies.

Assigning VLP contigs to probable microbial hosts is an ongoing challenge. Given a metagenomic sequence sample of viral genomes, say from stool, and a metagenomic analysis of the bacterial taxa present, how do you know who goes with who? Three approaches provide provisional annotation [10, 11, 13–15]. 1) On rare occasions, a VLP contig will closely resemble a database virus with a known host, allowing straightforward attribution. 2) Occasionally a VLP contig will have a reasonably close match to a continuous sequence in a bacterial genome, supporting the idea that the VLP contig corresponds to a temperate phage infecting the queried bacteria. 3) If CRISPR spacers present in a bacterial genome match sequences in a VLP contig from the same environment, it seems reasonable to infer that the virus can infect the CRISPR-containing bacteria. Unfortunately, application of the three methods still usually specifies phage/host relationships for a small minority of VLP contigs in a metagenomic sample. Several groups are developing further methods for use with this problem [69].

Viruses that grow on human cells instead of bacterial cells are typically rare in stool virome samples from healthy subjects. Viral lineages detected include single stranded DNA viruses such as Anelloviruses, Circoviruses, and Parvoviruses, and double-stranded DNA viruses such as Adenoviruses and Papillomaviruses. For RNA viruses in health human stool, viruses of plants seem to predominate, and are inferred to be transients from food. In one memorable study, Pepper mild mottle virus was found to predominate in stool from subjects in California. Extensive detective work showed that the virus was in fact abundant in hot sauce, the apparent source [19].

All these inferences, of course, are greatly complicated by the fact that most genomes in a sample are from viruses that have never been studied. As we become more adept at interrogating the viral dark matter, our thinking on the above points will likely evolve.

### Virome of monozygotic twins and mothers

In one of the earliest comprehensive studies of the human gut virome, Gordon and colleagues [10] investigated the viral component of the human microbiome in healthy individuals using metagenomic sequencing of fecal samples from four pairs of adult female monozygotic twins and their mothers at three time points over a one year period. They found that prophages and temperate phages were abundant in the samples, including Podoviridae, Myoviridae and Siphoviridae families.

They predicted the hosts of the some of the identified VLP contigs using the approaches described above, and found them to be members of the phyla Firmicutes and Bacteroidetes. The majority of the virome was unique to each individual, familial relationships notwithstanding, and showed high inter-personal variability but negligible intra-personal variability over the time period studied. Over 95% of viral genotypes persisted over the one year sampling period [70], and a later study of one healthy adult individual over ~2.5 years showed ~80% persistence [13]. The above studies were ground-breaking, but still the authors could not annotate ~81% of reads, highlighting the importance of the viral dark matter.

### Virome and its response to diet

Gut bacteria are affected by diet [71, 72], so diet is expected to change the composition of phage communities as well. In one study of the dynamics of the human gut virome under a dietary intervention [15], Minot et al. studied fecal samples from six adults on either of two controlled diets for 10 days. Virus-like particles (VLPs) were purified from stool and sequenced, then reads assembled. The authors found each individual harbored a unique and stable virome over the 10 days, suggesting that gut phages are not acquired from food on daily time scales. Individuals on the same diet converged detectably in population composition, suggesting that diet did influence virome composition.

Gordon and colleagues studied [28] the development of the infant virome in healthy and malnourished twins in Malawi. Previous work [73] from the Gordon group had demonstrated that the cellular gut microbiota influences severe acute malnutrition (SAM), so the authors further investigated the role of virome. They sequenced VLPs in fecal samples from 8 pairs of monozygotic and dizygotic twins concordat for healthy growth and 12 twin pairs discordant for SAM over the first three years of life together with their mothers and siblings. The authors developed a machine learning algorithm on virome sequencing reads and identified age discriminatory viruses in healthy twins. They further compared these viruses with those identified from SAM discordant datasets and found phages and eukaryotic viruses belonging to Anelloviridae and Circoviridae families can discriminate discordant from healthy twin pairs. SAM was characterized by a virome community and as well as an immature microbiome. Even the apparently healthy child in the discordant pair had an immature virome, suggesting they may have increased risk for malnutrition. This virome signature was also present after standard therapeutic food therapy for malnutrition, suggesting monitoring the virome may help guide development of improved interventions.

In the sections below we turn to metagenomic studies of phage mobilization. We first review transfer of medically significant gene types between bacteria, then movement of whole viral communities between human individuals during fecal microbial transplantation.

### Transport and integration of medically important genes by phage

Temperate bacteriophage can transport genes between bacteria and install them in the bacterial genome by integration [74, 75]. These genes are then inherited like normal bacterial genes during DNA replication and cell division. Upon sensing of a suitable inducing signal such as DNA damage, the prophage can excise, replicate lytically, and release progeny capable of infecting new cells [76–81]. Thus, cells harboring prophages— “lysogens”—can show novel phenotypic characteristics resulting from expression of genes on prophages, some of which are medically relevant.

For example, phages are well known to transport toxin genes between bacterial cells [82–84]. Shiga toxin, cholera toxin, and numerous others are carried on temperate phage, so that transduction renders lysogenic bacteria toxin producers. Integration of the phage genome into the bacterial genome can take place via either phage-encoded integrases (shiga toxin) [84] or by hijacking host cell recombination machinery (cholera toxin) [83]. Virome studies are just beginning to report the global frequency of occurrence of such toxin genes in different environments [82]. Other gene types are also known to influence human health [25].

Less clear has been the extent to which antibiotic resistance genes have been transferred between bacteria via phage. Historically, phage transduction has been viewed as only a minor contributor to transmission of antibiotic resistance genes, with transformation and particularly conjugation mediating transfer to a far greater extent [75]. However, a recent metagenomic study suggested that phage commonly encode antibiotic resistance genes, and that in mice the frequency of antibiotic resistance genes on phage actually increases with antibiotic treatment [85]. This supported a disturbing model in which antibiotic treatment actually caused wholesale mobilization of resistance genes via phage.

However, a recent reanalysis of annotation methods suggested a technical explanation. If thresholds for annotating antibiotic resistance genes are excessively permissive, then many calls may be erroneous misattribution of genes with other functions. Enault et al. [86] carried out a careful comparison of annotation thresholds for calling antibiotic resistance genes, combined with functional tests, and suggested that in fact the thresholds used by Modi et al. were far too permissive, so that far fewer resistance genes were present than initially thought. Analysis of fully sequenced phage genomes yielded only four clear examples of well-supported antibiotic resistance genes [86]. More data in this area would be helpful, but it now seems that the original picture may have been correct, and phage are only rare carriers of antibiotic resistance genes.

It is also rare to find transposons integrated into phage genomes. Thus, a major piece of the apparatus important for transmissible antibiotic resistance is again rare in phage. Possibly this is due to packaging efficiency: viral capsids can incorporate only a certain amount of nucleic acid, and lengthening viral genomes by transposon insertion may result in genomes that are incorporated relatively inefficiently.

### Movement of phage between humans during fecal microbial transplantation

Fecal microbiota transplant has been successful in treatment of relapsing *Clostridium difficile* (*C. difficile*) infections [87]. FMT treatment appears to work by restoring a more normal anaerobic gut community, though measurements typically show that the new communities in patients are complex mixtures of strains from donor, recipient, and new acquisition [88]. The general behavior and possible contribution of the virome in FMT is just starting to be investigated.

Chehoud et al. [31] sequenced the virome from a case series in which feces from a single donor was used to treat three children with ulcerative colitis (UC). Recipients received multiple FMT treatments over a 6 to 12 weeks’ period. Possible transient clinical benefit was observed [89]. The authors sequenced donor and recipient VLP samples, and assembled contigs from the reads. Multiple donor viral contigs were detected in the donor and in each recipient. Up to 42 donor contigs were detected in recipients, some annotating to specific bacteriophage families, documenting extensive transfer of phage communities. Chehoud et al. also investigated features associated with preferential transfer of viruses from donors to recipients, and found signatures of lysogeny in the transmitted viruses--the two most frequently transferred gene types were associated with temperate phage replication, and *Siphoviridae*, the group including lambda, were transferred with high efficiency. This led to the proposal that lysogeny may exist in part to assist in phage dispersal between environments.

More recently, Zuo and colleagues [65] investigated the role of the virome in FMT treatment for *C. difficile* infection. They sequenced the virome from 24 subjects with *C. difficile*, of whom 9 were treated with FMT and 5 received standard care with antibiotics, and 20 healthy controls. They found that before treatment patients with *C. difficile* had a higher abundance of phages from *Caudiovirales* (tailed bacteriophages) but lower diversity, richness and evenness compared to healthy controls. Following FMT treatment, subjects who responded showed an increased abundance of *Caudiovirales* contigs from the donor compared with those who did not respond. This raises the intriguing possibility that phage may be involved in successful FMT, possibly consistent with a published pilot study in which fecal extracts lacking bacteria were potentially effective in treating Clostridium difficile infection [90].

## Conclusions

Recognition of the vast phage populations associated with humans prompts numerous questions on their biology. How many different kinds are there? What are their replication styles and rates? How do genes transported by phage influence bacterial phenotypes relevant to human health? Most broadly, how do phage affect human welfare?

We are starting to see proposals for associations between large groups of phages and specific human disease. For example, Caudovirales have been associated with human inflammatory bowel disease in some [17] but not all [91] studies. The Caudovirales are a large and heterogenous order—it seems surprising that they should be behaving similarly as a group, but mechanisms have been proposed to explain this [17]. Similarly, as mentioned above, Caudovirales abundance has been associated with success in fecal microbial transplantation [65], another intriguing idea that awaits confirmation in further data sets.

Phage-mediated DNA mobilization no doubt also strongly influences human-associated communities and thereby human health. Phage were recently shown to move DNA between gut Salmonella strains in mice in response to induction by reactive oxygen species [92]. Likely myriad phage in gut move between bacterial species in response to further inducing agents characteristic of the gut environment, many of which are likely to be unidentified so far. It will be valuable to characterize transfer in more detail in human-associated settings. Finally, movement of whole phage populations between individuals are just starting to be studied, with initial focus on FMT due to the experimental accessibility.

Recent work provides a new window on an old problem, which is the role of lysogeny in phage ecology [93]. Rohwer and colleagues have suggested [1] a “Piggyback-the-Winner” model, where lysogeny is favored at high microbial density. This is in contrast with the earlier “Kill-the-Winner” model [94, 95], which suggests that once a microbial host achieves a high density, it is increasingly preferentially targeted by a predator phage which replicates on the predominant strain. The abundant strain then decreases in relative proportion, resulting in increased microbial diversity of the prey community, thus emphasizing the importance of lytic growth. Piggyback-the-winner suggests that phage actually replicate more efficiently in many environments as a prophage installed in successful bacteria. Recent studies [93, 96] have also highlighted the role of lysogeny in mediating resistance to phage superinfections via phage-encoded phage resistance genes encoded on prophage. In addition, as mentioned above, studies of FMT suggest that lysogeny may also assist in phage dispersal. Thus, contemporary virome studies lead us to think about the role of lysogeny in several new ways.

We end with a conjecture on the nature of the viral dark matter [16]. Why is such a large fraction of phage DNA sequence unlike any previously studied? One idea is that genomes of DNA phage are under pressure to change their primary sequences in response to pressure from restriction endonucleases and CRISPR systems. Ongoing host-virus competition, played out at a replication rate as fast as 20 min per cycle, will drive high rates of sequence diversification. If this is then multiplied over the estimated 10^31^ viral particles on Earth, it becomes easier to understand how phage have diversified to an extreme degree. A corollary is that despite the rapid drift in the primary DNA sequence, protein structure and function may be more conserved. In a few cases there are multiple X-ray structures for different phage proteins that carry out conserved functions, allowing assessment of their resemblance. For the phage repressor and Cro proteins, which are important in regulating lysogeny, the DNA sequences from lambda, 434 and P22 have little resemblance (median identity 34%), and even less resemblance at the protein level (median identity 17%) [97]. However, the encoded proteins show generally similar structures, dominated by the helix-turn-helix DNA binding motif and supporting alpha-helical secondary structures [98–102]. If this is generalizable, then perhaps once phage protein structures and functions are better worked out, understanding the viral dark matter will become less daunting.

## Abbreviations

*C. difficile*: Clostridium Difficile
CRISPR: Clustered interspersed short palindromic repeats
CsCl: Cesium chloride
EM: Electron Microscopy
FMT: Fecal Microbiota Transplant
SAM: Severe acute malnutrition
VLP: Virus-like particle
